# Use of High-Field Electron Injection into Dielectrics to Enhance Functional Capabilities of Radiation MOS Sensors

**DOI:** 10.3390/s20082382

**Published:** 2020-04-22

**Authors:** Dmitrii V. Andreev, Gennady G. Bondarenko, Vladimir V. Andreev, Alexander A. Stolyarov

**Affiliations:** 1 Bauman Moscow State Technical University, the Kaluga branch, 2 Bazhenov str., 248000 Kaluga, Russia; dmitrii_andreev@bmstu.ru (D.V.A.); a.a.stolyarov@bmstu.ru (A.A.S.); 2 National Research University Higher School of Economics, 20 Myasnitskaya str., 101000 Moscow, Russia; gbondarenko@hse.ru

**Keywords:** MOS sensor, RADFET, gate dielectric, ionization radiation, high-field, absorbed dose

## Abstract

The paper suggests a design of radiation sensors based on metal-oxide-semiconductor (MOS) structures and p-channel radiation sensitive field effect transistors (RADFET) which are capable to function under conditions of high-field tunnel injection of electrons into the dielectric. We demonstrate that under these conditions, the dose sensitivity of the sensor can be significantly raised, and, besides, the intensity of radiation can be monitored in situ on the basis of determining the ionization current arising in the dielectric film. The paper proposes the model allowing to make a quantitative analysis of charge effects taking place in the radiation MOS sensors under concurrent influence of ionization radiation and high-field tunnel injection of electrons. Use of the model allows to properly interpret results of the radiation control. In order to test the designed sensors experimentally, we have utilized γ-rays, α-particle radiation, and proton beams. We have acquired experimental results verifying the enhancement of function capabilities of the radiation MOS sensors when these have been under high-field injection of electrons into the dielectric.

## 1. Introduction

Nowadays in the field of medicine, space devices, nuclear power, etc., metal-oxide-semiconductor (MOS) sensors of a radiation are widely utilized [1,2,3,4,5,6,7,8,9,10,11,12,13,14,15]. Most frequently, these sensors are based on p-channel MOSFET (radiation sensitive field effect transistors (RADFET) sensors). In a gate dielectric of the sensors, when these are under an ionization radiation, a positive charge is accumulated and rising of density of surface states takes place [1,2,3,4,5,9]. As a result, one observes a change of threshold voltage of RADFET sensors. Using reference dependencies acquired at earlier stage, one calculates an absorbed dose on the basis of threshold voltage shift [1,9,10,11,12,13,14,15]. One of the main techniques to raise the sensitivity of RADFET sensors is an applying of positive voltage to the transistor gate [1,4,9,14]. Electric field in the dielectric film, caused by the applied voltage, initiates separation of charges, generated by radiation ionization, and increases yield of holes which avoided recombination. Then these holes travel to the interface with silicon where a fraction of these become trapped resulting in threshold voltage shift [1,9,13]. Thus, a presence of positive voltage at the gate of RADFET sensor causes rising of its dose sensitivity. At that, dose sensitivity increases with rising of the gate voltage. However, voltage level, applied to the gate, is usually limited by values at which the electric field in the gate dielectric does not exceed 1–2 MV/cm [1,5,9,12]. This specifies by possibility of arising of degradation processes influencing onto precision and efficiency of the sensors and, besides, by difficulties of maintaining of sensor stable functioning under such conditions. A method of dose sensitivity rising of RADFET sensors by using the dielectric of a higher thickness [3,5,8,11] has numerous limitations. In order to form this dielectric, the method of chemical vapor deposition is commonly used which results in the dielectric film of a lower quality with respect to the thermal oxidation method. As a consequence, the dielectric film of a higher thickness can degrade under relatively low fields [16] and it is more difficult to achieve the field and thermal stability of sensors based on these dielectric films. Therefore, developing radiation MOS sensors, which are capable to function under high fields, including a condition of electron injection into the dielectric, and new techniques to monitor radiation is a subject of great practical importance, which implies the rising dose sensitivity and extending of functional capabilities of the sensors.

In the work we have designed radiation MOS sensors which have been capable to function under high-field injection of electrons into the dielectric. Functioning characteristics of the sensors under high fields have been studied. In addition, we have tested these sensors at monitoring of characteristics of proton beams, α-particle radiation, and γ-rays.

The rest of this paper is organized as follows. Section 2 describes the design and manufacturing technology of the utilized sensors and technique of the experiment. Section 3 presents experimental results and discussion of these. Finally, Section 4 summarizes the study and includes potential application of the sensors.

## 2. Materials and Methods

**Figure 1:**
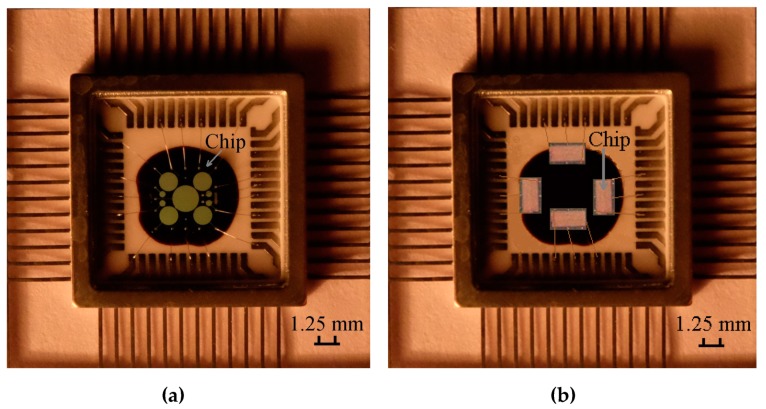
Photography of the radiation metal-oxide-semiconductor (MOS) sensors based on the MOS capacitors (**a**) and four p-channel MOSFETs (radiation sensitive field effect transistors (RADFETs)) (**b**) enclosed into a metal-ceramic package.

In the paper we utilize two types of radiation MOS sensors. The first type is implemented on the basis of MOS capacitors with different areas manufactured on a single semiconductor crystal [15]. The second type of the sensors is the p-channel MOSFET with channel length of 6 μm and width of 700 μm. Both types of the sensors are manufactured in full compliance with CD4000 manufacturing technology of integrated circuits. As the base semiconductor wafers, we utilized n-type silicon doped with phosphorus with resistivity of 4.5 Ω × cm and <100> crystallographic orientation. Source/drain regions of the p-channel MOSFET were formed by ion implantation of boron with subsequent thermal impurity distribution at temperature of 1000 °C. The gate dielectric is the SiO_2_ film with thickness of 100 nm which is formed by thermal oxidation of silicon at temperature of 1000 °C in atmosphere of dry oxygen with adding of 3% gas-phase HCl. Using the magnetron deposition and subsequent photolithography the aluminum gates were formed. The MOS capacitors have different areas of the top electrode which are 10^−4^ cm^2^, 10^−3^ cm^2^, 10^−2^ cm^2^, and 2 × 10^−2^ cm^2^. After formation of the gates, we implemented thermal annealing of the semiconductor wafers at temperature of 450 °C in nitrogen atmosphere. After that, for convenience of contacting an enclosing of crystals into a 48 leads metal-ceramic package was realized. Photography of the utilized MOS sensors are presented in Figure 1.

Thus, both types of sensors have the same dielectric film formed in common technological process. This dielectric film allows to implement the high-field injection of electrons into the dielectric for used sensors [15,16,17,18,19,20,21] what is the main distinctive feature of these. In order to initiate the mode of high-field electron injection we utilized an experimental setup realized by means of precision source/measuring device of constant current (voltage) NI PXIe-4135 which was controlled by the special program implemented in NI LabVIEW. The experimental setup allows to realize the charge injection into the dielectric for the mode of maintaining of both constant current and constant voltage. In addition, it allows to monitor the change of charge state of MOS sensors using technique suggested in Andreev et al. [22] and Andreev et al. [23]. All experiments were realized under applying of positive bias voltage to the sensor gate. When implementing the mode of electron high-field injection, the source and drain of the RADFET were connected to the bulk. In order to obtain additional information about changing of charge state of MOS sensors, we monitored threshold voltage of the transistors and measured C-V characteristics of the capacitors.

The irradiation by protons was implemented by means of experimental setup based on a particle accelerator. That allowed to use proton beams with energies of 150–500 keV. One pulse by the accelerator provided a proton fluence of 10^10^ cm^2^. Density of proton current was (1–4) × 10^−8^ A/cm^2^. That value allowed to avoid heating of the observed samples.

In order to study an influence of α-particles on the MOS sensors, we treated the samples by radiation of ^239^Pu source. α-particles flow rate was 10^10^ s^−1^ × cm^−2^. As a rule, the experimental samples were placed at a distance of few millimeters from the radiation source. In order to irradiate the samples by γ-rays we used ^60^Co source. 

## 3. Results and Discussion

### 3.1. Physical Effects in MOS Sensors

**Figure 2:**
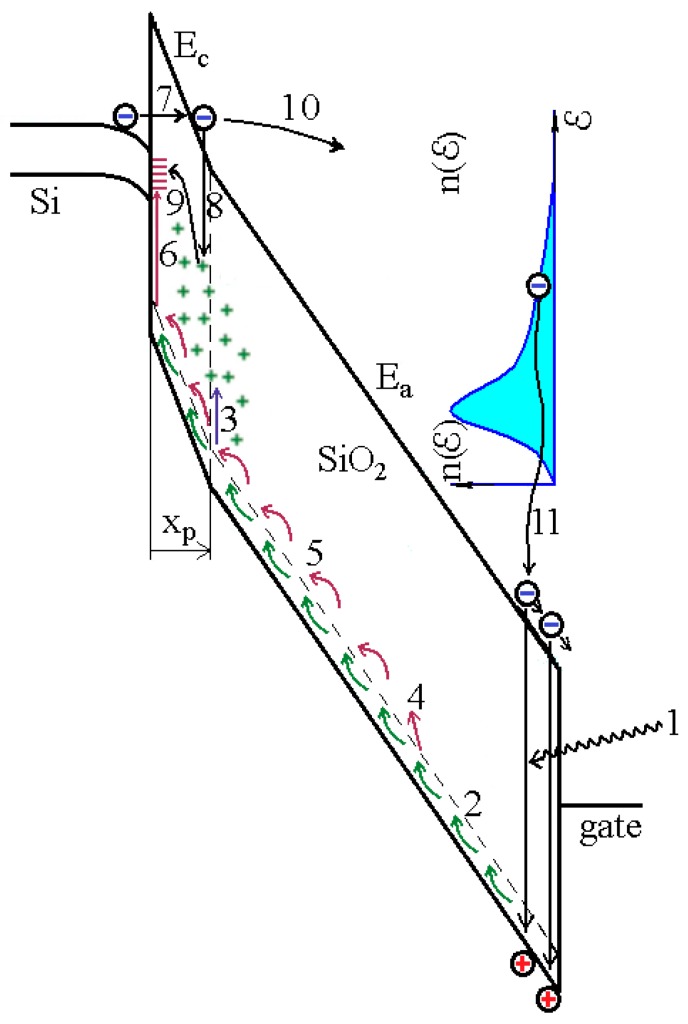
Band diagram of the MOS structure demonstrating main charge effects taking place under concurrent influence by radiation and high-field tunnel injection of electrons by Fowler–Nordheim: 1—creation of electron-hole pairs by radiation; 2—hole transport; 3—capturing of holes on traps in SiO_2_ at its interface with silicon; 4—hydrogen liberation; 5—hydrogen transport; 6—interaction of hydrogen and defects with subsequent creation of surface states; 7—Fowler–Nordheim high-field injection of electrons; 8—annihilation of a fraction of trapped holes when interacting with injected electrons; 9—generation of surface states as a result of annihilation of holes; 10—transport and heating of injected electrons in the conduction band of SiO_2_; 11—thermalization of hot electrons with subsequent creation of holes.

In order to raise dose sensitivity of the MOS sensors we used the mode of electron high-field injection into the gate dielectric. Under the mode, electric fields in the dielectric film significantly increase and these stimulate the accumulation of higher values of positive charge at the same radiation dose. However, when the sensor is under the mode, besides charge effects caused by radiation ionization (Figure 2) and discussed in detail in Schwank et al. [24], Fleetwood et al. [25], and Oldham et al. [26], it is essential to take into consideration the following charge effects [27,28]: additional generation of positive charge as a result of thermalization of injected electrons (Figure 2, process 11), annihilation of a fraction of positive charge caused by interaction with injected electrons (Figure 2, process 8), generation of additional surface states which takes place because of annihilation of a positive charge fraction and injected electrons (Figure 2, process 9). In accordance with the model suggested in Andreev et al. [27] and Andreev et al. [28], we use the following set of equations to describe sensor functioning when it is under concurrent influence of ionizing radiation and high-field injection of electrons:

- in order to calculate density of holes accumulated in the gate dielectric under concurrent influence of radiation and high-field tunnel injection of electrons we apply the following equation:

(1) $$q\frac{dp}{dt}=\left(J_{\mathrm{inj}}\times \alpha +J_{\mathrm{rad}}\right)\times \sigma_{p}\times \left(N_{p}-p\right)-J_{\mathrm{inj}}\times \sigma_{\mathrm{ep}}\times p;$$

- in order to compute a value of the cathode field we use Fowler–Nordheim equation for current density [17,18,19]:

(2) $$J_{\mathrm{inj}}=AE_{c}{}^{2}\exp\left(-\frac{B}{E_{c}}\right);$$

- in order to calculate current density initiated by ionization radiation we apply the following equation [24,27,28]:

(3) $$J_{\mathrm{rad}}=q\times Y\left(E\right)\times K_{g}\times d_{\mathrm{ox}}\times I_{\mathrm{rad}};$$

- equation of shift of voltage across MOS structure under electron injection from silicon when constant current is maintained:

(4) $$\Delta V_{I}\left(+\right)=\frac{q}{\varepsilon \varepsilon_{0}}\left[p\left(d_{\mathrm{ox}}-x_{p}\right)\right];$$

where α—coefficient of high-field ionization in the SiO_2_ film at thermalization of hot injected electrons; σ_ep_—cross-section of injected electrons by filled hole traps (at annihilation of a fraction of positive charge) and the cross-section depends on the field as following σ_ep_ = *b*_0_ × *E*^−3^, where *b*_0_—model parameter [17]; *q*—electron charge; σ_p_—cross-section of hole traps; *N*_p_—density of hole traps; *A* = 1.54 × 10^−6^ × *m*_0_/m^*^ × ϕ_B_^−1^ [A/V^2^] и *B* = 6.83 × 10^7^ × *m*_0_/m^*^ × ϕ_B_^3/2^ [V/cm]—constants of tunnel Fowler–Nordheim injection [16]; *m*_0_ and m*—mass of electron in vacuum and effective mass of electron in the dielectric, accordingly; ϕ_B_—height of potential barrier at the injecting interface; *Y*(*E*)—charge yield under irradiation (a fraction of holes which avoided recombination); *K*_g_—a value of electron-hole pairs per dose unit and SiO_2_ volume (8 × 10^12^ cm^−3^ × rad^−1^) [24]; *d*_ox_—thickness of the oxide; *I*_rad_ = d*D*/d*t*—irradiation intensity (rad/s); *D*—irradiation dose (rad); εε_0_—dielectric permittivity; *x*_p_—the location of positive charge centroid of holes trapped in the SiO_2_ film (with respect to the Si/SiO_2_ interface).

The paper discusses radiations which have relatively high intensity at low duration of these. As a result of this, the generation of surface states is not of high rate and we assume that positive charge, accumulated in the dielectric film as a result of ionization radiation, is the main information parameter characterizing radiation characteristics. In accordance with the statement a shift of threshold voltage of the RADFET sensor Δ*V*_th_ is equal to the shift of voltage across MOS structure, when constant current flowing is maintained, and that shift is described by Equation (4), i.e., Δ*V*_th_ = Δ*V*_I_(+) [22,23]. This assumption is in a good agreement with experimental results.

Experimental results, demonstrated in the paper, show that the surface area of the MOS capacitor virtually does not influence on radiation effects taking place in the gate dielectric of the sensor. We utilized the MOS sensors of different surface area in order to ease the involving of required modes of high-field injection. Most experimental results, demonstrated in the paper, have been obtained by utilization of the MOS sensors with gate area of 10^−3^ cm^2^ and 10^−2^ cm^2^ and by utilization of the RADFET sensors for which charge effects have had similar quantitative characteristics as compared to the MOS capacitors under concurrent influence by radiation and high-field injection of electrons.

### 3.2. Proton Irradiation

**Figure 3:**
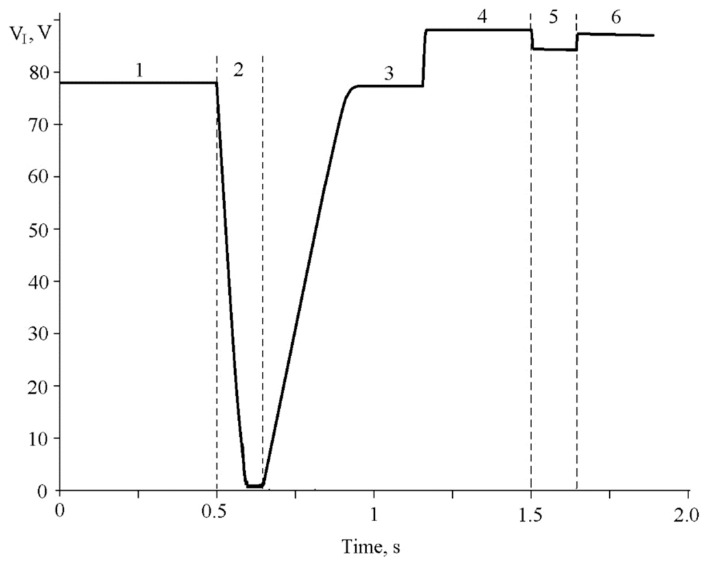
Time dependence of voltage across MOS structure measured when constant current with density of 10^−6^ A/cm^2^ (Sections 1,2,3) or 5 × 10^−5^ A/cm^2^ (Sections 4–6) flows through the dielectric under irradiation by one pulse of proton beam (Sections 2 and 5).

We have studied an influence of proton irradiation on MOS structures, which have been under high-field injection of electrons into the dielectric, utilizing the particle accelerator. Before the MOS structures, placed in the accelerator chamber, to be irradiated, we applied to these a pulse of constant current and reached transition of these to the mode of Fowler–Nordheim high-field tunnel injection of electrons from silicon into the dielectric. We have irradiated the MOS structures, which has been under the injection, by short pulses of the proton beams. During all experiment we have controlled time dependence of voltage across the MOS structure (Figure 3). Figure 3 demonstrates that in Section 1 the structure has been switched to the mode of high-field tunnel injection by a pulse of current with density of 10^−6^ A/cm^2^. Then in Section 2 it has been irradiated by the proton beam with energy of 500 keV and fluence of 10^10^ cm^−2^.

When applying the pulse of constant current with density of *J*_0_ to the MOS structure, we can use the following equation to determine sum of densities of currents flowing through the dielectric film [27]:

(5) $$J_{0}=J_{c}+J_{\mathrm{inj}}+J_{\mathrm{rad}};$$

where *J*_c_ = *C* (d*V*/d*t*)—density of capacitive current; *C*—capacitivity of MOS structure; *J*_inj_—density of high-field tunnel injection current of electrons; *J*_rad_—density of ionization current initiated in MOS structure because of radiation.

If density of ionization current, initiated as a result of influence by radiation, is equal to *J*_rad_ and higher than *J*_0_, then one would observe discharge of capacity of MOS structure down to voltages close to zero (Figure 3, Section 2). Value of ionization current in the high-voltage section of discharge of MOS structure capacitance, calculated using Equation (5) from Figure 3, Section 2, is 2 × 10^−5^ A/cm^2^.

In Section 4 (Figure 3) we rose the density of current, applied to the MOS structure, up to 5 × 10^−5^ A/cm^2^. Thus, in Section 5 (Figure 3), when proton radiation is presented and it is analog to the value in Section 2, *J*_rad_ becomes lower than *J*_0_ and voltage, at which the structure switches to high-field tunnel injection of electrons, also becomes lower because of diminishing of injection current density (Equation (5)) in comparison with Sections 4 and 6 in which radiation is not presented.

We have monitored value of positive charge, accumulated in the gate dielectric after irradiation, by measuring an increment of voltage across the MOS sensor before and after influence of radiation (Figure 3, Sections 1 and 3 or 4 and 6, accordingly). Hence, by measuring ΔV_I_(+) or shift of threshold voltage of RADFET sensor, we can calculate integral absorbed dose of ionization radiation [1,2,3,4,5,9]. An advantage of the RADFET sensors in comparison with the MOS capacitors is the simpler technique to monitor the change of charge state of the dielectric when it is under radiation treatment. This technique is based on measuring of threshold voltage shift of the FET [1,2,3,4,5,6,7,8,9,10,11,12,13,14]. For the MOS capacitors based sensors, we monitor an accumulation of positive charge in the gate dielectric by using whether C-V measurements or voltage shift across the structure when it is under high-field injection of electrons under the mode of constant current flowing (Equation (4)). However, sensors on the basis of MOS capacitors have higher capacitance with respect to the RADFETs and that simplifies high-field injection of charge into the dielectric by low current densities. A capability to measure C-V characteristics of the capacitor based MOS sensors gives an ability to obtain more complete information about change of surface state density what is important when monitoring low-intensity radiations. Therefore, in order to implement the comprehensive monitoring, it is necessary to utilize the sensors of both types as on the basis of the RADFET as on the basis of the MOS capacitors.

Under the mode of flowing of constant current through the dielectric, the initiation of the ionization current, when the sensor is under radiation, results in lowering of injection and/or capacitive current, flowing through the dielectric, and that, in its turn, causes change of time dependence of voltage across the MOS structure (Equation (5)). Therefore, by monitoring change of time dependence of voltage across the MOS structure and using Equations (1)–(5), one can calculate the value of ionization current caused by radiation. In that case, the sensitivity of the MOS sensor will be few times higher than it is for usual technique of monitoring of radiation intensity using a RADFET sensor for which that parameter is calculated by measuring threshold voltage of a RADFET sensor [3,11]. Change of threshold voltage of a RADFET sensor is caused by the accumulation of positive charge in the gate dielectric at the Si/SiO_2_ interface which takes place because of capturing on traps of a not significant fraction of holes created when radiating. Thus, in situ measurement of ionization current, initiated in the dielectric film when radiating, significantly raises sensitivity of the sensor.

### 3.3. α-Particle Irradiation

**Figure 4:**
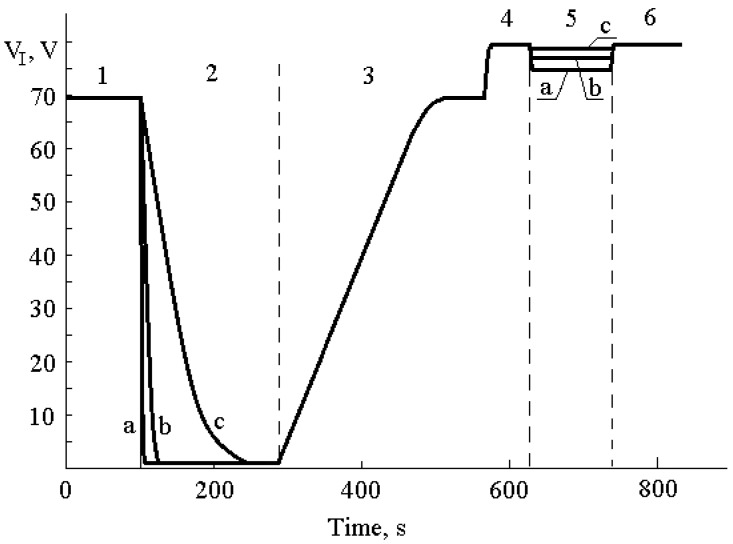
Time dependence of voltage across the MOS structure when constant current of different densities flows through dielectric: 1,2,3—10^−8^ A/cm^2^; 4,5,6—10^−6^ A/cm^2^; for Sections 2 and 5 we realized irradiation by α-particles when the radiation source was in varying distances from the MOS sensor: **a**—4 mm; **b**—15 mm; **c**—30 mm.

Figure 4 shows experimental results, illustrating the influence of α-particles on MOS structures when constant current pulse with density of 10^−8^–10^−6^ A/cm^2^ is applied to the sample. Section 1 in Figure 4 corresponds to the high-field tunnel injection of electrons from the silicon substrate by constant current with density of 10^−8^ A/cm^2^. Then we were placing the source of α-particles in a varying distance from the gate of the MOS sensor (Figure 4, Sections 2 and 5 correspond to the radiation). As Figure 4 demonstrates, widening the distance between source and MOS sensor gate results in lowering of ionization current initiated in the dielectric film. We calculated a value of the ionization current by analyzing time dependence of voltage across the structure using Equations (1)–(5). In Figure 4 (Section 3), all the current flowing through the MOS structure is capacitive and rising rate of voltage, in accordance with Equation (5), is totally defined by the value of constant current flowing through the sample. In order to lower the duration of Sections 3 and 4, one can implement charging of the MOS structure capacitance by the current of higher density using the technique suggested in Andreev et al. [23] and in that case the duration of Sections 3 and 4 could be a few seconds. The experimental data, shown in Figure 4, have been measured, as demonstrated in the figure, in order to simplify interpretation of the results and illustrate the operation principles of the suggested sensor.

Taking into consideration the experimental data, demonstrated in Figure 3 and Figure 4, we can suggest the following technique to monitor the intensity of radiation. Initially we switch the MOS sensor to the mode of high-field tunnel injection of electrons at a low density of the injection current and monitor a value of the ionization current on the basis of lowering of the voltage, applied to the sensor gate to maintain the constant current mode. If the influence by radiation under the mode results in termination of the injection current flowing (in case the ionization current, caused by the radiation, is higher than the injection current) then by the rate of MOS sensor capacitance discharge using Equations (1)–(5) we can calculate the value of the ionization current. Next in order to implement a more precise measurement of a density of the ionization current, we switch the MOS sensor to the mode of high-field injection, at flowing of the injection current of the constant density not much higher than the ionization current, and in this section we one more time calculate the density of ionization current on the basis of lowering of the voltage at the gate of the MOS sensor.

### 3.4. Measurement of Absorbed Dose

**Figure 5:**
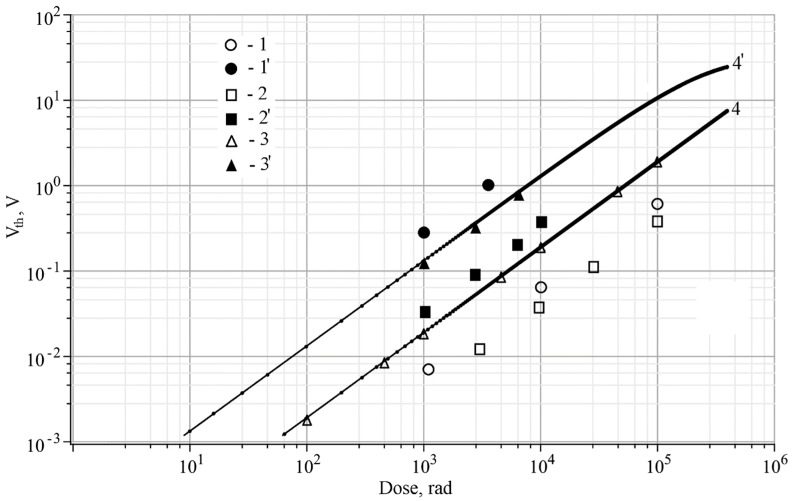
Experimental (1,1′,2,2′,3,3′) and theoretical (4,4′) dependencies of threshold voltage shift of the MOS sensor (Δ*V*_th_) on radiation dose in case of proton influence (1,1′), α-particles (2,2′), and γ-rays (3,3′,4,4′): 1,2,3,4—with no bias at the gate; 1′,2′,4′—under high-field injection by current of 10^−6^ A/cm^2^; 3′—at positive bias at the gate (*E*_a_ = 5 MV/cm).

Figure 5 demonstrates experimental and theoretical (these are calculated using the model suggested) dose dependencies of threshold voltage shift of the MOS sensors (Δ*V*_th_) under radiation of protons, α-particles, and γ-rays. The RADFET sensors have similar dose characteristics with respect to sensors on the basis of MOS capacitors. Figure 5 shows that when under the mode of high-field injection of electrons the sensor has maximal sensitivity, however, at that, the dose range can be significantly reduced. The reducing of the MOS sensor dose range is caused by partial filling of hole traps by positive charge generated by high-field tunnel injection of electrons and by acceleration of degradation processes in the gate dielectric occurring because of high-field injection. The annihilation of a fraction of positive charge and injected electrons, as it has been shown in Andreev et al. [27] and Andreev et al. [28], can result in rising of amount of surface states at the Si/SiO_2_ interface, and it can accelerate degradation processes cause by restructuring of the interface and the dielectric film.

Evaluative calculations of total ionizing dose (TID), which have been implemented on the basis of dependencies used in Ravotti [7] and Haran et al. [29], when irradiating by protons in Sections 2 and 5 of Figure 3, give a value of about 10^3^ rad. These experimental results, obtained from data presented in Figure 3, are demonstrated in Figure 5 (symbols 1′). When irradiating by the α-particles in Section 5 of Figure 4, TID is about 10–100 rad which value depends on distance between the MOS sensor and the radiation source. Figure 5 shows experimental data obtained at radiation intensity of 1 rad/s.

Figure 5 demonstrates that under the influence of protons and α-particles one observes higher rise of the MOS sensor sensitivity when switching to the high-field injection mode and that probably caused by more noticeable increasing of the charge yield for these sections with respect to the γ-rays irradiation [27]. Thus, in order to obtain high dose sensitivity of the sensor under the γ-rays radiation, it is enough to apply voltage providing creation of electric field which is slightly lower than injection field. In that study, when γ-rays influencing, we maintained electric field in the dielectric with the value of 5 MV/cm. Accumulation of positive charge in the gate dielectric of MOS structure during irradiation results in lowering of the potential barrier at the Si/SiO_2_ interface and decreasing of the anode field (Figure 2) at the most part of the dielectric film. Hence, when the sample is under irradiation, by lowering the voltage, applied to the gate of the MOS sensor, by a value calculated by Equation (4), one can reach a maintaining of the electric field at constant value at the most part of the dielectric film and an avoiding of the mode of high-field injection into the dielectric. Data 3′ in Figure 5 correspond to this measurement mode and these provide the dose sensitivity similar to the high-field injection mode (curve 4′) and, at the same time, avoiding of the injection mode does not reduce sensor lifetime.

Thus, utilization of the MOS sensors under the mode of high-field injection of charge into the dielectric requires the additional analysis of charge effects taking place in the dielectric film and it is required to take influence of these effects into consideration when processing experimental data and choosing operation modes of the sensors [27,28]. For this analysis one can use the model, suggested in the paper, taking into consideration parameters specific to sensors chosen.

Utilization of the MOS sensors under the high-field injection of electrons into the dielectric can raise the dose sensitivity of the sensor more than ten times (Figure 5). However, use of the sensor under the conditions requires involving of the model of charge state change of the dielectric, suggested in the paper, to separate the radiation portion of positive charge. The high-field injection of electrons into the gate dielectric can cause significant degradation processes in the gate dielectric and, as a result, the dose range of the sensor can be drastically reduced. Thus, that is appropriate to use the high-field injection mode at low values of the dose range (up to 10^4^ rad) for cases when it is essential to obtain maximum dose sensitivity of the sensor as, for example, it is necessary for medical applications. In the most practical cases we consider as appropriate to utilize sensors, suggested in the paper, under the high fields, which are few lower (5%–10% lower) than injection fields, at that correcting the value of voltage applied to the sensor gate in accordance with the electric field induced by the positive charge accumulated in the dielectric. In that case, we succeeded in raising dose sensitivity of the sensor in few times in comparison with common mode of its utilization and, at that, we almost do not reduce its lifetime.

RADFET sensors with SiO_2_ dielectric films of the same thickness, as we utilize in the paper, were studied in detail in Yilmaz et al. [8], Pejović [9], Siebel et al. [12], and Ristic et al. [13]. When an electric bias is not applied to the gate and when the bias is 5 V, the sensors, described in Yilmaz et al. [8], Pejović [9], and Ristic et al. [13], have similar dose sensitivities to characteristics demonstrated in Figure 5. In Yilmaz et al. [8], Pejović [9], Siebel et al. [12], and Ristic et al. [13], authors do not investigate utilization of RADFET sensors under high fields apparently because of concern in degradation of these and absence of a physical model characterizing charge effects taking place in the MOS sensors under high fields especially in case when high-field injection of electrons into the dielectric is presented. Hence, manufacturing of the MOS sensors which are suggested in the paper and capable to function stably under high-field injection of electrons into the dielectric, and developing of a physical model characterizing charge effects taking place in the gate dielectric at concurrent influence of radiation and high-field injections, give a capability to raise the dose sensitivity of the sensors a few times in comparison with RADFET sensors described in literature and having similar structure parameters [8,9,12,13]. The designed sensors and techniques of monitoring of the radiation characteristics have a variety of applications where high dose sensitivity is important and/or there is a necessity to measure the radiation intensity such as in medicine, space applications, nuclear power, etc.

## 4. Conclusions

The paper suggests the design of radiation sensors based on the MOS structures and p-channel RADFET with the thermal SiO_2_ film. These sensors are capable to function under high-electric field including the case of high-field injection of electrons into the dielectric. We have demonstrated that under the mode of flowing of constant current through the dielectric, the initiation of ionization current, when radiation has been presented, has resulted in lowering of injection and/or capacitive current also flowing through the dielectric and that, in turn, has caused the changing of time dependence of voltage across MOS structure. By monitoring this voltage change we can calculate the value of ionization current caused by radiation. In that case, the dose sensitivity of the MOS sensor would be a few times higher than it is for the common technique to monitor a radiation intensity utilizing a RADFET sensor which allows to compute that parameter by measuring a change of threshold voltage of a RADFET.

We have demonstrated that in order to raise the dose sensitivity of the MOS sensors, one could utilize these under high fields in the gate dielectric, including cases when these fields have caused injection of electrons. We suggest the model allowing to implement the quantitative analysis of charge effects taking place in the MOS sensors of radiation under concurrent influence of both ionization radiation and high-field injection of electrons. Use of the model allows to correctly interpret results of radiation monitoring when the sensor is under the described modes. That is appropriate to use the high-field injection mode at low values of the dose range (up to 10^4^ rad) for cases when it is essential to obtain maximum dose sensitivity of the sensor as, for example, it is necessary for medical applications.

Experimental data obtained when analyzing influence of the protons, α-particles, and γ-rays verify the enhancement of the functional capabilities of the radiation MOS sensors in the case where these are under the mode of high-field injection of electrons into the dielectric.

## References

[1] Holmes-Siedle A., Adams L. (1986). RADFET: A review of the metal-oxide-silicon devices as dosimeters use of integrating. Radiat. Phys. Chem..

[2] Damulira E., Yusoff M.N.S., Omar A.F., Taib N.H.M. (2019). A Review: Photonic Devices Used for Dosimetry in Medical Radiation. Sensors.

[3] Kulhar M., Dhoot K., Pandya A. (2019). Gamma Dose Rate Measurement Using RadFET. IEEE Trans. Nucl. Sci..

[4] Camanzi B., Holmes-Siedle A.G. (2008). The race for new radiation monitors. Nat. Mater..

[5] Pejovica S.M., Pejovic M.M., Zivanovic M. (2019). Small dose effect in RADFET with thick gate oxide. Appl. Radiat. Isot..

[6] Zhang C., Hasan S.M.R. (2019). A New Floating-gate Radiation Sensor and Readout Circuit in Standard Single-Poly 130-nm CMOS Technology. IEEE Trans. Nucl. Sci..

[7] Ravotti F. (2018). Dosimetry Techniques and Radiation Test Facilities for Total Ionizing Dose Testing. IEEE Trans. Nucl. Sci..

[8] Yilmaz E., Kahraman A., McGarrigle A.M., Vasovic N., Yegen D., Jaksic A. (2017). Investigation of RadFET response to X-ray and electron beams. Appl. Radiat. Isot..

[9] Pejović M.M. (2017). Processes in radiation sensitive MOSFETs during irradiation and post irradiation annealing responsible for threshold voltage shift. Radiat. Phys. Chem..

[10] Pikhay E., Roizin Y., Nemirovsky Y. (2017). Ultra-Low Power Consuming Direct Radiation Sensors Based on Floating Gate Structures. J. Low Power Electron.

[11] Andjelkovic M.S., Ristic G.S., Jaksic A.B. (2015). Using RADFET for the real-time measurement of gamma radiation dose rate. Meas. Sci. Technol..

[12] Siebel O.F., Pereira J.G., Souza R.S., Ramirez-Fernandez F.J., Schneider M.C., Galup-Montoro C. (2015). A very-Low-Cost dosimeter based on the off-The-Shelf CD4007 MOSFET array for in vivo radiotherapy applications. Radiat. Meas..

[13] Ristic G.S., Vasovic N.D., Kovacevic M., Jaksic A.B. (2011). The sensitivity of 100 nm RADFETs with zero gate bias up to dose of 230 Gy(Si). Nucl. Instrum. Methods Phys. Res. B.

[14] Lipovetzky J., Holmes-Siedle A., Inza M.G., Carbonetto S., Redin E., Faigon A. (2012). New Fowler-Nordheim Injection, Charge Neutralization, and Gamma Tests on the REM FT300 RADFET Dosimeter. IEEE Trans. Nucl. Sci..

[15] Andreev V.V., Bondarenko G.G., Andreev D.V., Akhmelkin D.M. Sensors Based on MIS Structures for Study of Ionization Radiations. Proceedings of the Moscow Workshop on Electronic and Networking Technologies (MWENT).

[16] Strong A.W., Wu E.Y., Vollertsen R., Suñé J., Rosa G.L., Rauch S.E., Sullivan T.D. (2009). Reliability Wearout Mechanisms in Advanced CMOS Technologies.

[17] Arnold D., Cartier E., DiMaria D.J. (1994). Theory of high-field electron transport and impact ionization in silicon dioxide. Phys. Rev. B.

[18] Palumbo F., Wen C., Lombardo S., Pazos S., Aguirre F., Eizenberg M., Hui F., Lanza M. (2019). A Review on Dielectric Breakdown in Thin Dielectrics: Silicon Dioxide, High-k, and Layered Dielectrics. Adv. Funct. Mater..

[19] Wu E.Y. (2019). Facts and Myths of Dielectric Breakdown Processes—Part I: Statistics, Experimental, and Physical Acceleration Models. IEEE Trans. Electron. Devices.

[20] Andreev D.V., Bondarenko G.G., Andreev V.V., Maslovsky V.M., Stolyarov A.A. (2017). Modification of MIS Devices by Irradiation and High-Field Electron Injection Treatments. Acta Phys. Pol. A.

[21] Andreev D.V., Bondarenko G.G., Andreev V.V., Maslovsky V.M., Stolyarov A.A. (2019). Modification of MIS Devices by Radio-Frequency Plasma Treatment. Acta Phys. Pol. A.

[22] Andreev V.V., Bondarenko G.G., Maslovsky V.M., Stolyarov A.A., Andreev D.V. (2015). Control current stress technique for the investigation of gate dielectrics of MIS devices. Phys. Status Solidi. C.

[23] Andreev V.V., Maslovsky V.M., Andreev D.V., Stolyarov A.A. Method of stress and measurement modes for research of thin dielectric films of MIS structures. Proceedings of the International Conference on Micro- and Nano-Electronics.

[24] Schwank J.R., Shaneyfelt M.R., Fleetwood D.M., Felix J.A., Dodd P.E., Paillet P., Ferlet-Cavrois V. (2008). Radiation Effects in MOS Oxides. IEEE Trans. Nucl. Sci..

[25] Fleetwood D.M. (2018). Evolution of Total Ionizing Dose Effects in MOS Devices with Moore’s Law Scaling. IEEE Trans. Nucl. Sci..

[26] Oldham T.R., McLean F.B. (2003). Total Ionizing Dose Effects in MOS Oxides and Devices. IEEE Trans. Nucl. Sci..

[27] Andreev V.V., Maslovsky V.M., Andreev D.V., Stolyarov A.A. Charge effects in dielectric films of MIS structures being under high-field injection of electrons at ionizing radiation. In Proceeding of the International Conference on Micro- and Nano-Electronics.

[28] Andreev D.V., Bondarenko G.G., Andreev V.V., Stolyarov A.A. (2019). Simulation of charge processes in dielectric films of MIS structures at simultaneous influence by ionization and high-Field injection of electrons. Procedia Manuf..

[29] Haran A., Murat M., Barak J. (2008). Charge Yield and Track Structure Effects on Total Ionizing Dose Measurements. IEEE Trans. Nucl. Sci..

